# Dose-Response Association of Low-Intensity and Nondaily Smoking With Mortality in the United States

**DOI:** 10.1001/jamanetworkopen.2020.6436

**Published:** 2020-06-03

**Authors:** Maki Inoue-Choi, Carol H. Christensen, Brian L. Rostron, Candace M. Cosgrove, Carolyn Reyes-Guzman, Benjamin Apelberg, Neal D. Freedman

**Affiliations:** 1 Metabolic Epidemiology Branch, Division of Epidemiology and Genetics, National Cancer Institute, National Institutes of Health, Bethesda, Maryland; 2 Office of Science, Center for Tobacco Products Food and Drug Administration, Silver Spring, Maryland; 3 United States Census Bureau, Suitland, Maryland; 4 Tobacco Control Research Branch, Behavioral Research Program, Division of Cancer Control and Population Sciences, National Cancer Institute, National Institutes of Health, Bethesda, Maryland

## Abstract

**Importance:**

An increasing proportion of US smokers smoke at low intensity and not every day. Some nondaily smokers have always had this pattern, whereas others previously smoked daily. The effect of reducing the level of smoking from daily to nondaily smoking and the dose response at low smoking levels are poorly understood.

**Objective:**

To evaluate risk of all-cause and cause-specific mortality among nondaily and daily cigarette smokers, by cigarettes per month, years after reducing from daily to nondaily smoking, and years since quitting.

**Design, Setting, and Participants:**

A prospective cohort study using harmonized data from multiple cycles of the Tobacco Use Supplements to the Current Population Survey (TUS-CPS), linked to the National Death Index, were analyzed during the period from 2018 to 2020. Adults completed the 1992-1993, 1995-1996, 1998-1999, 2000, 2001-2002, 2003, 2006-2007, or 2010-2011 TUS-CPS. Cigarette smokers were classified as daily or nondaily users; current nondaily smokers were further categorized by whether they previously smoked every day.

**Main Outcomes and Measures:**

Hazard ratios (HRs) and 95% CIs for risks of mortality vs never smoking. Age was the underlying time metric, adjusted for sex, race/ethnicity, education, survey year, and household income.

**Results:**

Among 505 500 participants (aged 18-103 years), approximately 47 000 deaths occurred. The median number of cigarettes smoked per month was 600 (interquartile range, 300-600) (20 cigarettes per day [interquartile range, 10-20 cigarettes per day]) for daily cigarette smokers and 40 (interquartile range, 15-90) for lifelong nondaily smokers. Nevertheless, both current daily (HR, 2.32; 95% CI, 2.25-2.38) and lifelong nondaily (HR, 1.82; 95% CI, 1.65-2.01) smokers had higher all-cause mortality risks than never smokers. Associations were observed for 6 to 10 cigarettes per month and increased with greater-intensity use. Nondaily smokers who previously smoked every day had lower mortality risks than daily smokers, with similar HRs after 10 or more years of nondaily smoking as lifelong nondaily smokers (HR vs never smokers, 1.73; 95% CI, 1.56-1.92). Yet, their risks were higher than former smokers who quit 10 or more years before (HR vs never smokers, 1.18; 95% CI, 1.15-1.22).

**Conclusions and Relevance:**

Although reducing smoking from daily to nondaily was associated with decreased mortality risk, cessation was associated with far greater benefit. Lifelong nondaily smokers have higher mortality risks than never smokers, even among those smoking 6 to 10 cigarettes per month. Thus, all smokers should quit, regardless of how infrequently they smoke.

## Key Points

**Question:**

What is the association of reducing from daily to nondaily cigarette smoking with mortality risks?

**Findings:**

In a prospective cohort study of 505 500 nationally representative US adults, daily smokers had 2.32 times higher mortality risk, and lifelong nondaily smokers had 1.82 times higher mortality risk, than never smokers; significant associations were observed for 6 to 10 cigarettes per month and increased with higher-intensity use. Risks decreased when smokers reduced from daily to nondaily smoking, yet the benefits of cessation were far larger.

**Meaning:**

Nondaily smokers have substantially higher mortality risks than never smokers, even if they smoke just a few cigarettes per month.

## Teaser

This cohort study evaluates the risk of all-cause and cause-specific mortality among nondaily and daily cigarette smokers, by cigarettes per month, years after reducing from daily to nondaily smoking, and years since quitting.

## Introduction

Cigarette smoking results in an estimated 480 000 premature deaths in the United States and 7 million deaths worldwide each year.^1,2^ In the US, the prevalence of cigarette smoking has decreased dramatically owing to a wide range of public health programs. In 2018, 13.7% of US adults (34.2 million) smoked cigarettes, a 34.4% decrease from 2005 (20.9% [45.1 million adults]).^3,4^

In addition to the decreasing prevalence of smoking, an increasing number of smokers smoke at low intensity (<10 cigarettes per day) or on just some days of the month (nondaily). Among US current smokers who reported smoking in the past 30 days, the proportion of nondaily smokers increased from 19.2% in 2005 to 25.0% in 2018.^3,4^ The proportion of low-intensity daily smokers also increased, from 16.4% in 2005 to 25.0% in 2016.^3^ Nondaily smokers may differ substantively from daily smokers, both with regard to nicotine dependence and in reasons for smoking.^5,6^ In light of continued policy, regulatory, and societal changes and the emergence of other tobacco products, the numbers of occasional and low-intensity cigarette smokers are expected to increase.

There is a common perception among smokers that nondaily smoking poses little or no harm.^7^ Nevertheless, emerging studies indicate that lifelong nondaily smokers have an approximately 1.7 times higher mortality risk and a 5-year shorter median life expectancy compared with never smokers.^8^ Nondaily smokers also benefit from smoking cessation.^8^ Numerous questions remain, however. Among nondaily smokers, the dose reponse is poorly understood. The association of changing from daily to nondaily smoking with mortality is also unknown. Such data will inform the potential contribution of nondaily smoking to the worldwide tobacco epidemic.

To answer these key questions, we harmonized multiple cycles of the Tobacco Use Supplement to the Current Population Survey (TUS-CPS) linked to the National Longitudinal Mortality Survey (NLMS), leveraging detailed information on smoking patterns among both nondaily and daily cigarette smokers with a large US representative sample size.

## Methods

### Study Population

The TUS-CPS is a National Cancer Institute/National Institutes of Health–sponsored national survey for tobacco use, administered as part of the US Census Bureau’s Current Population Survey every few years since 1992-1993. The US Centers for Disease Control and Prevention cosponsored the 2001-2002, 2003, and 2006-2007 waves, and the US Food and Drug Administration, Center for Tobacco Products cosponsored the 2014-2015 and 2018-2019 waves. In each cycle, approximately 240 000 adults in eligible households among the civilian, noninstitutionalized US population received questionnaires. Approximately 64% of respondents completed the questionnaire by telephone, and approximately 36% completed the questionnaire via in-person interviews.

The TUS-CPS is a cross-sectional survey,^9^ but a subset of the TUS-CPS has been linked to mortality data from the National Death Index (currently through 2011), as part of the NLMS. The NLMS consists of US Census Bureau data from the CPS and Annual Social and Economic Supplements that have been linked with death certificate data. The present analysis includes the subset of participants in the NLMS who completed the TUS surveys.

The human subject internal review board of the National Institutes of Health determined this study to be exempt because data are not identifiable. All analyses were performed by a statistician of the US Census Bureau during the period from 2018 to 2020. We rounded all weighted and unweighted numbers following the disclosure avoidance guidelines by the US Census Disclosure Review Board to protect study participants’ confidentially. This study is reported according to the Strengthening the Reporting of Observational Studies in Epidemiology (STROBE) reporting guideline.

### Tobacco Use

We combined tobacco use data from the 1992-1993, 1995-1996, 1998-1999, 2000, 2001-2002, 2003, 2006-2007, and 2010-2011 TUS-CPS questionnaires into a single data set for analyses. From approximately 705 000 individuals in the TUS-CPS NLMS linked data from these years, we excluded 34 000 who died before the start of follow-up or were not eligible for mortality linkage, 131 000 who reported having smoked less than 100 cigarettes in their lives, 1000 with incomplete smoking information, and 8300 with unknown race/ethnicity. We also excluded individuals who were younger than 18 years of age or older than 103 years of age because smoking-related deaths are unlikely to occur at very old ages and to exclude extreme survivors. After these exclusions, the analysis included approximately 505 500 individuals.

We defined ever cigarette smokers as individuals who reported smoking 100 cigarettes or more in their lives. Current smokers reported smoking cigarettes in the past 30 days, either every day (daily smokers) or on some days of the month (nondaily smokers). Nondaily smokers were stratified into those who previously smoked daily (nondaily, previous-daily smokers) and those who never smoked daily (lifelong nondaily smokers). We categorized former smokers, who reported not smoking in the past 30 days, into former daily smokers and former nondaily smokers.

We assessed detailed smoking patterns, including number of days smoked in the past 30 days, number of cigarettes smoked on days smoked, age started smoking, and age at cessation. We computed the number of cigarettes smoked per day or in the past 30-day month and categorized current daily smokers (≤1, >1-2, >2-10, >10-20, >20-30, and >30 cigarettes per day) and nondaily smokers (≤5, 6-10, 11-30, 31-60, and >60 cigarettes per month). We stratified current nondaily smokers who had previously smoked daily by number of years since reducing the level of smoking from daily to nondaily (<2, 2-9, and ≥10 years). We also stratified former daily and nondaily smokers by number of years since cessation (<2, 2-4, 5-9, and ≥10 years). Never cigarette smokers were the referent group in all analyses.

### Follow-up and Mortality Outcomes

We followed participants for mortality from survey administration through the end of 2011, and approximately 47 000 deaths occurred during follow-up. In addition to all-cause mortality, we defined underlying cause of death according to the *International Statistical Classification of Diseases and Related Health Problems, Tenth Revision* (*ICD-10*) codes as follows: all cancers (*ICD-10* codes C00-C97), smoking-related cancers (lip, oral cavity, and pharynx [*ICD-10* codes C00-C14]; esophagus, stomach, colon and rectum, liver, and pancreas [*ICD-10* codes C15, C16, C18-C21, C22, and C25]; larynx, trachea, bronchus, and lung [*ICD-10* codes C32 and C33-C34]; cervix, kidney, bladder [*ICD-10* codes C53, C64-C65, and C67], and myeloid leukemia [*ICD-10* code C92]), circulatory diseases (*ICD-10* codes I00-I09, I20-I25, I26-I28, I29-I51, I60-I69, I70, and I71, I72-I78), cerebrovascular disease (*ICD-10* codes I60-I69), respiratory disease including influenza, pneumonia, and chronic obstructive pulmonary disease (COPD) (*ICD-10* codes J10-J11, J12-J18, J40-J42, J43, and J44), and all other causes.

### Statistical Analysis

We used Cox proportional hazards regression to estimate hazard ratios (HRs) and 95% CIs using age as the underlying time metric. We adjusted final models for sex, race/ethnicity (non-Hispanic white, non-Hispanic black, Hispanic, and other), education (<high school, high school, some college, and college), TUS-CPS survey year (1992-1993, 1995-1996/1998-1999, 2000/2001-2002/2003, 2006-2007, and 2010-2011), and ratio of standardized income to the poverty level (<50%, 50% to <100%, 100% to <200%, 200% to <400%, and ≥400%) based on literature review. We evaluated and confirmed the proportional hazards assumption by visual assessment of −log-log plots of survival function by time (age). Survival curves were created from Cox regression models.

Relevant NLMS sampling weights were set to the noninstitutional US population size, appropriately accounting for the cluster sampling design of the original surveys. We reweighted sampling weights for differences in cohort size and incorporated in all models.

We performed a sensitivity analysis for all-cause mortality excluding participants who reported ever using other tobacco products (cigar, pipe, and smokeless tobacco). We conducted all analyses using Unix SAS, version 9.4 (SAS Institute Inc), 2-tailed statistical tests, and *P* < .05 as the threshold for statistical significance.

## Results

Among approximately 505 500 participants (235 000 men and 270 500 women; 18-103 years of age), approximately 47 000 deaths occurred. Approximately 96 600 (19.1%) were current cigarette smokers, 109 800 (21.7%) were former smokers, and 299 200 (59.2%) were never smokers. Of current smokers, approximately 78 870 (81.6%) reported smoking every day, and 17 730 (18.4%) reported smoking nondaily. Among nondaily smokers, 42.9% had previously smoked every day, 35.5% were lifelong nondaily smokers, and 21.6% did not report whether they had previously smoked every day or not.

Daily smokers smoked a median of 20 cigarettes per day (interquartile range [IQR], 10-20 cigarettes per day) (600 cigarettes per month [IQR, 300-600 cigarettes per month]) (Table 1). Nondaily smokers typically smoked on 15 days (IQR, 6-20 days for lifelong nondaily smokers; IQR, 10-20 days for nondaily, previous-daily smokers) of the month and smoked far fewer cigarettes per month than daily smokers (lifelong nondaily smokers: 40 cigarettes per month [IQR, 15-90 cigarettes per month]; nondaily, previous-daily smokers: 75 cigarettes per month [IQR, 30-150 cigarettes per month]). Nondaily and daily smokers both started smoking at a median age of 17 to 18 years. Among never cigarette smokers, 8.1% reported ever using other tobacco products (cigar, pipe, and smokeless tobacco), whereas approximately 20% to 23% of current cigarette smokers reported ever using other tobacco products. E-cigarettes and similar devices were not assessed in the surveys included in this analysis.

**Table 1.** Demographic Characteristics and Cigarette Smoking Patterns by Smoking Status Among 505 500 Adults in the 1992-1993, 1995-1996, 1998-1999, 2000, 2001-2002, 2003, 2006-2007, and 2010-2011 TUS-CPS NLMS Cohort^a^^,^^b^

Characteristic or pattern	Never smoker	Current smoker			Former smoker	
		Daily	Nondaily, previous-daily	Lifelong nondaily	Daily	Never daily
No. (%)^c^	299 200 (59.2)	78 870 (15.6)	7607 (1.5)	6292 (1.2)	75 450 (14.9)	16 020 (3.2)
Age, y, % (95% CI)						
18-24	13.3 (13.2-13.4)	10.4 (10.1-10.6)	9.1 (8.3-9.9)	16.5 (15.4-17.6)	2.2 (2.1-2.4)	4.6 (4.2-5.0)
25-34	19.2 (19.0-19.3)	19.7 (19.4-20.1)	22.2 (21.1-23.2)	28.9 (27.6-30.2)	9.0 (8.8-9.2)	14.1 (13.5-14.8)
35-44	20.4 (20.3-20.6)	24.3 (23.9-24.6)	24.2 (23.1-25.4)	23.8 (22.6-25.1)	14.9 (14.6-15.2)	17.9 (17.2-18.5)
45-54	17.5 (17.3-17.6)	22.6 (22.3-23.0)	19.6 (18.5-20.6)	16.2 (15.2-17.2)	20.0 (19.7-20.4)	19.1 (18.4-19.8)
≥55	29.6 (29.4-29.8)	23.0 (22.7-23.4)	24.9 (23.8-26.1)	14.7 (12.6-15.7)	53.9 (53.5-54.3)	44.3 (43.4-45.2)
Sex, % (95% CI)						
Male	42.4 (42.1-42.6)	52.8 (52.4-53.2)	46.3 (45.0-47.6)	52.4 (51.0-53.9)	52.9 (52.5-53.4)	50.2 (49.3-51.1)
Female	57.7 (57.4-57.9)	47.2 (46.8-47.6)	53.7 (52.4-55.0)	47.6 (46.2-9.1)	47.1 (46.6-47.5)	49.8 (48.9-50.8)
Race/ethnicity, % (95% CI)						
Non-Hispanic						
White	71.9 (71.7-72.0)	82.2 (81.9-82.5)	78.3 (77.2-79.4)	62.8 (61.4-64.2)	88.4 (88.1-88.6)	82.1 (81.5-82.8)
Black	11.9 (11.8-12.1)	9.8 (9.6-10.1)	11.6 (10.7-12.5)	17.2 (16.0-18.3)	6.1 (5.9-6.2)	7.1 (6.7-7.6)
Hispanic	10.1 (10.0-10.2)	4.3 (4.2-4.5)	6.1 (5.5-6.6)	14.2 (13.3-15.1)	3.1 (3.0-3.2)	7.3 (6.9-7.6)
Other	6.1 (6.0-6.2)	3.7 (3.5-3.8)	4.0 (3.5-4.5)	5.9 (5.2-6.5)	2.5 (2.3-2.6)	3.4 (3.1-3.7)
Education, % (95% CI)						
<High school	14.3 (14.1-14.4)	20.4 (20.0-20.7)	14.5 (13.6-15.5)	17.2 (16.1-18.2)	14.0 (13.8-14.3)	13.0 (12.4-13.6)
High school	29.2 (29.0-29.4)	42.5 (42.1-42.9)	34.0 (32.8-35.3)	32.5 (31.2-33.9)	33.5 (33.1-33.9)	29.0 (28.2-29.9)
Some college	26.6 (26.4-26.8)	26.5 (26.2-26.9)	30.7 (29.5-32.0)	30.4 (29.1-31.8)	27.8 (27.4-28.2)	26.5 (25.7-27.3)
College	29.9 (29.7-30.1)	10.6 (10.4-10.9)	20.7 (19.6-21.8)	19.9 (18.7-21.1)	24.6 (24.3-25.0)	31.4 (30.6-32.3)
Cigarette smoking pattern, median (IQR)						
Age when started smoking, y	NA	17 (15-19)	18 (16-20)	18 (16-21)	17 (15-19)	17 (12-19)
No of days smoked in past 30 d	NA	30 (30-30)	15 (10-20)	15 (6-20)	NA	NA
No. of cigarettes smoked per past 30 d	NA	600 (300-600)	75 (30-150)	40 (15-90)	NA	NA
Ever used other tobacco products	8.1 (8.0-8.2)	19.8 (19.5-20.2)	23.0 (21.8-24.1)	20.9 (19.7-22.1)	26.8 (26.5-27.2)	23.0 (22.2-23.8)

Abbreviations: DRB, Disclosure Review Board; IQR, interquartile range; NA, not applicable; NLMS, National Longitudinal Mortality Survey; TUS-CPS, Tobacco Use Supplement to the Current Population Survey.

^a^ US Census Bureau’s DRB release number CBDRB-FY19-262.

^b^ Weighted data; numbers were weighted and rounded to 4 significant digits following the disclosure avoidance guidelines by the US Census Bureau’s DRB.

^c^ Percentages were calculated including participants who did not report ever smoking daily information.

Lifelong nondaily smokers were younger (69% were <45 years) than daily smokers (54% were <45 years) and nondaily, previous-daily smokers (56% were <45 years). Compared with daily smokers, lifelong nondaily smokers were more likely to be from racial/ethnic minority groups (non-Hispanic black, Hispanic, or other); the percentages of those from racial/ethnic minority groups were 37.2% for lifelong nondaily smokers, 21.7% for nondaily, previous-daily smokers, and 17.8% for current daily smokers. More current nondaily smokers (lifelong nondaily smokers, 19.9%; nondaily, previous-daily smokers, 20.7%) had a college education than current daily smokers (10.6%).

Lifelong nondaily smokers had 1.82 times higher all-cause mortality risk than never smokers (95% CI, 1.65-2.01) (Table 2). Risks were higher among daily smokers (HR, 2.32; 95% CI, 2.25-2.38) and comparable for nondaily, previous-daily smokers (HR, 1.93, 95% CI, 1.80-2.07). Similar associations were observed among men and women and when excluding ever users of other tobacco products. Mortality risks among lifelong nondaily smokers were lower among Hispanic individuals (HR, 1.18; 95% CI, 0.73-1.89) than non-Hispanic white individuals (HR, 1.81; 95% CI, 1.59-2.05) and non-Hispanic black individuals (HR, 1.88; 95% CI, 1.56-2.25), although the number of deaths among Hispanic smokers was low. Greater associations for daily smoking were found higher among non-Hispanic white individuals (HR, 2.44; 95% CI, 2.37-2.52) than non-Hispanic black individuals (HR, 1.76; 95% CI, 1.62-1.90) and Hispanic individuals (HR, 1.90; 95% CI, 1.61-2.25).

**Table 2.** All-Cause and Cause-Specific Mortality Risks by Cigarette Smoking Status Among Current Smokers^a^

Mortality	Never smoker	Current smoker		
		Daily	Nondaily, ever daily	Lifelong nondaily
**All-cause mortality**				
All				
Deaths, No.^b^	20 500	8500	800	350
HR (95% CI)^c^	1 [Reference]	2.32 (2.25-2.38)	1.93 (1.80-2.07)	1.82 (1.65-2.01)
Excluding ever users of other tobacco products				
Deaths, No.	19 000	7000	650	300
HR (95% CI)	1 [Reference]	2.31 (2.25-2.38)	1.91 (1.76-2.06)	1.78 (1.60-1.98)
Men				
Deaths, No.	7300	4800	400	200
HR (95% CI)	1 [Reference]	2.28 (2.19-2.37)	1.85 (1.67-2.05)	1.92 (1.66-2.22)
Women				
Deaths, No.	13 500	3700	450	200
HR (95% CI)	1 [Reference]	2.33 (2.24-2.42)	2.01 (1.83-2.22)	1.72 (1.50-1.98)
Non-Hispanic white				
Deaths, No.	16 500	7100	650	250
HR (95% CI)	1 [Reference]	2.44 (2.37-2.52)	2.03 (1.87-2.20)	1.81 (1.59-2.05)
Non-Hispanic black				
Deaths, No.	2300	850	100	100
HR (95% CI)	1 [Reference]	1.76 (1.62-1.90)	1.63 (1.36-1.95)	1.88 (1.56-2.25)
Hispanic				
Deaths, No.	1100	250	30	20
HR (95% CI)	1 [Reference]	1.90 (1.61-2.25)	1.51 (0.92-2.48)	1.18 (0.73-1.89)
Non-Hispanic other				
Deaths, No.	750	300	30	20
HR (95% CI)	1 [Reference]	2.30 (1.94-2.72)	1.88 (1.18-3.01)	1.11 (0.60-2.04)
**Cause-specific mortality**				
All cancer				
Deaths, No.^b^	3700	2500	200	80
HR (95% CI)^c^	1 [Reference]	3.14 (2.98-3.32)	2.46 (2.14-2.83)	1.76 (1.41-2.21)
Smoking-related cancer				
Deaths, No.	2000	2100	200	50
HR (95% CI)	1 [Reference]	4.88 (4.57-5.22)	3.65 (3.11-4.27)	2.16 (1.64-2.86)
Lung cancer				
Deaths, No.	450	1400	100	30
HR (95% CI)	1 [Reference]	13.96 (12.50-15.59)	10.22 (8.28-12.60)	5.64 (3.89-8.18)
Cardiovascular disease				
Deaths, No.	7200	2200	200	100
HR (95% CI)	1 [Reference]	1.92 (1.82-2.02)	1.60 (1.40-1.83)	1.69 (1.40-2.04)
Cerebrovascular disease				
Deaths, No.	1600	350	50	20
HR (95% CI)	1 [Reference]	1.51 (1.33-1.70)	1.57 (1.16-2.12)	1.45 (0.93-2.26)
Respiratory disease^d^				
Deaths, No.	1100	900	90	30
HR (95% CI)	1 [Reference]	5.98 (5.42-6.58)	5.66 (4.60-6.94)	3.46 (2.38-5.02)
Other cause				
Deaths, No.	10 500	3300	300	200
HR (95% CI)	1 [Reference]	1.75 (1.67-1.84)	1.48 (1.30-1.68)	1.82 (1.57-2.11)

Abbreviations: DRB, Disclosure Review Board; HR, hazard ratio.

^a^ US Census Bureau’s DRB release numbers CBDRB-FY19-262, CBDRB-FY19-358, CBDRB-FY19-391, CBDRB-FY19-262, and CBDRB-FY19-303.

^b^ Unweighted numbers of deaths were rounded following the disclosure avoidance guidelines by the US Census Bureau’s DRB: if the number is less than 15, report it; if the number is between 15 and 99, round to the nearest 10; if the number is between 100 and 999, round to the nearest 50; if the number is between 1000 and 9999, round to the nearest 100; if the number is between 10 000 and 99 999, round to the nearest 500; if the number is between 100 000 and 999 999, round to the nearest 1000; and if the number is 1 000 000 or more, round to 4 significant digits.

^c^ Adjusted for sex, race/ethnicity (non-Hispanic white, non-Hispanic black, Hispanic, and non-Hispanic other), education (<high school, high school, some college, and college), survey year (1992-1993, 1995-1996/1998-1999, 2000/2001-2002/2003, 2006-2007, and 2010-2011), and ratio of standardized income to the poverty level (<50%, 50% to <100%, 100% to <200%, 200% to <400%, ≥400%, and missing).

^d^ Respiratory disease includes chronic obstructive pulmonary disease, influenza, and pneumonia.

Nondaily smoking was associated with a number of different causes of death (Table 2). Relative to never smokers, mortality risks for a smoking-related cancer was 2.16 times (95% CI, 1.64-2.86) higher among lifelong nondaily smokers, 3.65 times (95% CI, 3.11-4.27) higher among nondaily, previous-daily smokers, and 4.88 times (95% CI, 4.57-5.22) higher among daily smokers. The HRs for lung cancer were 5.64 (95% CI, 3.89-8.18) among lifelong nondaily smokers, 10.22 (95% CI, 8.29-12.60) among nondaily, previous-daily smokers, and 13.96 (95% CI, 12.50-15.59) among daily smokers compared with never smokers. Mortality risks were also higher among lifelong nondaily smokers for all cancers (HR, 1.76; 95% CI, 1.41-2.21), cardiovascular disease (HR, 1.69; 95% CI, 1.40-2.04), respiratory disease (HR, 3.46; 95% CI, 2.38-5.03), and other causes of death (HR, 1.82; 95% CI, 1.57-2.11) than for never smokers.

**Figure 1.**
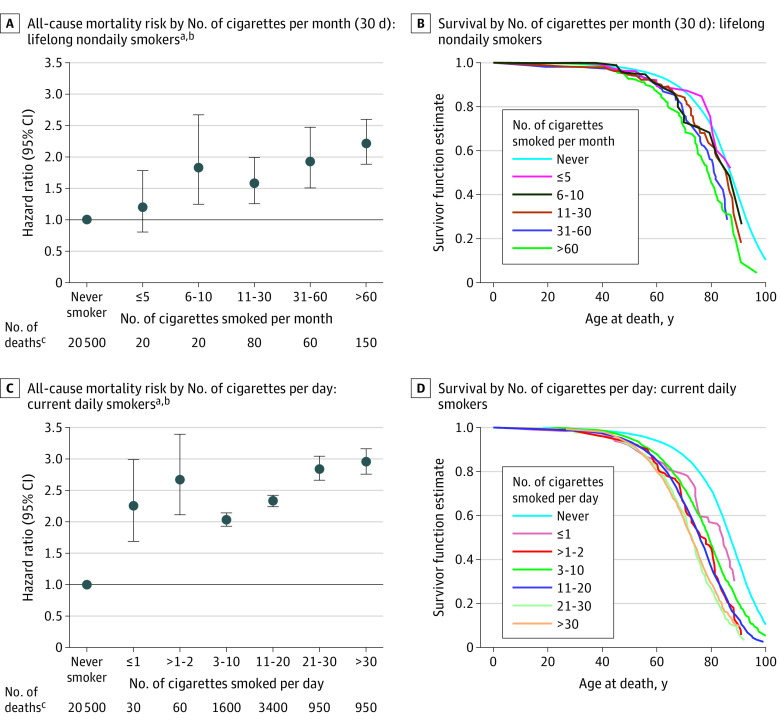
All-Cause Mortality Risk and Survival by Number of Cigarettes Smoked per Month (30 Days) or per Day ^a^US Census Bureau’s Disclosure Review Board (DRB) release numbers CBDRB-FY19-262 and CBDRB-FY20-CES004-014. ^b^Adjusted for sex, race/ethnicity (non-Hispanic white, non-Hispanic black, Hispanic, and non-Hispanic other), education (<high school, high school, some college, and college), survey year (1992-1993, 1995-1996/1998-1999, 2000/2001-2002, 2006-2007, and 2010-2011), and ratio of standardized income to the poverty level (<50%, 50% to <100%, 100% to <200%, 200% to <400%, ≥400%, and missing). ^c^Unweighted numbers were rounded following the disclosure avoidance guidelines by the US Census Bureau’s DRB: if the number is less than 15, report it; if the number is between 15 and 99, round to the nearest 10; if the number is between 100 and 999, round to the nearest 50; if the number is between 1000 and 9999, round to the nearest 100; if the number is between 10 000 and 99 999, round to the nearest 500; if the number is between 100 000 and 999 999, round to the nearest 1000; and if the number is 1 000 000 or more, round to 4 significant digits.

Among lifelong nondaily smokers, mortality risks increased in association with higher numbers of cigarettes per month (Figure 1). The HRs (95% CIs) for all-cause mortality were 1.18 (0.79-1.75) for 5 or less cigarettes per month, 1.80 (1.23-2.64) for 6 to 10 cigarettes per month, 1.56 (1.24-1.96) for 11 to 30 cigarettes per month, 1.91 (1.49-2.44) for 31 to 60 cigarettes per month, and 2.16 (1.83-2.54) for more than 60 cigarettes per month. A similar dose-response association was observed among current daily smokers, with increased risk observed even at less than 1 or 1 cigarette per day (HR, 2.23; 95% CI, 1.67-2.96). Current daily smokers who smoked more than 30 cigarettes per day had still higher mortality risks (HR, 2.94; 95% CI, 2.75-3.14). We also observed a shorter median survival with a higher number of cigarettes per month and per day among lifelong nondaily smokers and current daily smokers, respectively (Figure 1).

**Figure 2.**
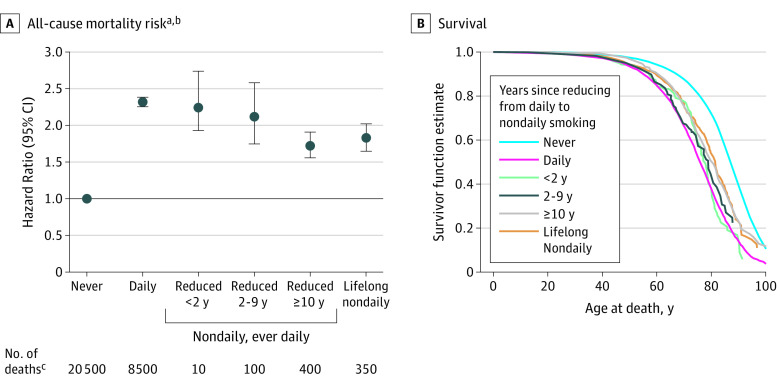
All-Cause Mortality Risk and Survival by Years Since Reducing From Daily to Nondaily Smoking Among Current Nondaily, Ever-Daily Smokers ^a^US Census Bureau’s Disclosure Review Board (DRB) release numbers CBDRB-FY19-26 and CBDRB-FY20-CES004-014. ^b^Adjusted for sex, race/ethnicity (non-Hispanic white, non-Hispanic black, Hispanic, and non-Hispanic other), education (<high school, high school, some college, and college), survey year (1992-1993, 1995-1996/1998-1999, 2000/2001-2002, 2006-2007, and 2010-2011), and ratio of standardized income to the poverty level (<50%, 50% to <100%, 100% to <200%, 200% to <400%, ≥400%, and missing). ^c^Unweighted numbers were rounded following the disclosure avoidance guidelines by the US Census Bureau’s DRB: if the number is less than 15, report it; if the number is between 15 and 99, round to the nearest 10; if the number is between 100 and 999, round to the nearest 50; if the number is between 1000 and 9999, round to the nearest 100; if the number is between 10 000 and 99 999, round to the nearest 500; if the number is between 100 000 and 999 999, round to the nearest 1000; and if the number is 1 000 000 or more, round to 4 significant digits.

Figure 2 depicts the mortality risks of reducing the level of smoking from daily to nondaily. A dose-dependent association was observed, whereby the lowest risk was observed among respondents who reduced their level of smoking from daily to nondaily 10 or more years ago (HR, 1.73; 95% CI, 1.56-1.92), similar to the risk among lifelong nondaily smokers. Similar patterns were observed when comparing median survival across these groups.

Among former daily smokers, mortality risks decreased in association with increasing years of cessation (Table 3). Relative to never smokers, the risk among those who quit less than 2 years ago (HR, 2.18; 95% CI, 2.03-2.35) was similar to the risk among current daily smokers (HR, 2.32; 95% CI, 2.25-2.38), whereas the lowest risk was observed among those who quit 10 or more years ago (HR, 1.18; 95% CI, 1.15-1.22). Similarly, there was evidence for an association with number of years since cessation among former nondaily smokers, although risk estimates were imprecise owing to a small number of deaths.

**Table 3.** All-Cause Mortality Risk by Years Since Cessation Among Former Daily and Nondaily Smokers Compared With Risk Among Never Smokers^a^

No. of years since quitting	Total No.^b^	Deaths, No.	HR (95% CI)^c^
Never smoker	281 000	20 500	1 [Reference]
Former daily smoker			
<2	5900	750	2.18 (2.03-2.35)
2 to <5	8900	1100	1.96 (1.84-2.08)
5 to <10	11 000	1500	1.69 (1.60-1.78)
≥10	44 500	7900	1.18 (1.15-1.22)
Former nondaily smoker			
<2	1100	50	1.20 (0.91-1.59)
2 to <5	1600	100	1.57 (1.29-1.90)
5 to <10	1900	100	1.36 (1.14-1.63)
≥10	10 500	1300	1.09 (1.04-1.15)

Abbreviations: DRB, Disclosure Review Board; HR, hazard ratio.

^a^ US Census Bureau’s DRB release number CBDRB-FY19-262.

^b^ Unweighted numbers were rounded following the disclosure avoidance guidelines by the US Census Bureau’s DRB: if the number is less than 15, report it; if the number is between 15 and 99, round to the nearest 10; if the number is between 100 and 999, round to the nearest 50; if the number is between 1000 and 9999, round to the nearest 100; if the number is between 10 000 and 99 999, round to the nearest 500; if the number is between 100 000 and 999 999, round to the nearest 1000; and if the number is 1 000 000 or more, round to 4 significant digits.

^c^ Adjusted for sex, race/ethnicity (non-Hispanic white, non-Hispanic black, Hispanic, and non-Hispanic other), education (<high school, high school, some college, and college), survey year (1992-1993, 1995-1996/1998-1999, 2000/2001-2002/2003, 2006-2007, and 2010-2011), and ratio of standardized income to the poverty level (<50%, 50% to <100%, 100% to <200%, 200% to <400%, ≥400%, and missing).

## Discussion

In a large nationally representative study, we observed that low-intensity and nondaily smoking was associated with substantially higher mortality risks than never smoking. Although mortality decreased for smokers who reduced their level of smoking from daily to nondaily, the benefits of cessation were far greater. Our analysis also provides insight into the dose response of low-intensity smoking. We observed increased mortality risks among nondaily smokers who smoked just 6 to 10 cigarettes per month, well under 1 cigarette per day, with higher mortality risks among those smoking additional amounts. The magnitude of these associations suggest that even lower levels of smoking may be associated with risk. Future studies with larger numbers of very low-intensity smokers will be informative.

The large sample size allowed us to systematically compare 2 groups of nondaily smokers. Although a majority of nondaily smokers used to smoke every day and at least some may be trying to quit, an additional set of nondaily smokers reported always having a nondaily smoking pattern. Lifelong nondaily smokers tended to be younger and smoke fewer cigarettes per month than those who had previously smoked every day, although both groups tended to have started smoking in their teenage years.

We were also able to investigate the association of reducing the level of smoking from daily to nondaily with mortality risks. Daily smokers who became nondaily smokers substantially reduced their monthly cigarette consumption, and their subsequent mortality risks decreased to those of lifelong nondaily smokers after 10 years of reduction. Nevertheless, their mortality risks remained substantially greater than both never and former smokers. Thus, our study provides important evidence on the benefits of smoking cessation beyond even substantial reductions in cigarette smoking.

Previous studies on the disease and mortality risks of nondaily cigarette smoking have been limited but are generally consistent with our findings. In a previous analysis of TUS-CPS NLMS data, current nondaily cigarette smokers had 1.60 times (95% CI, 1.52-1.69) higher all-cause mortality than never smokers.^10^ Similarly increased risks of all-cause mortality were reported among current occasional smokers in cohort studies in Finland (HR, 1.6; 95% CI, 1.3-2.1) and Norway (HR, 1.38, 95% CI, 1.08-1.76).^11,12^ However, these previous studies did not differentiate lifelong nondaily smokers from nondaily smokers who previously smoked daily. In the National Health Interview Survey, lifelong nondaily smokers had 1.72 times all-cause mortality (95% CI, 1.36-2.18) and 5-year shorter survival, on average, than never smokers,^8^ although the number of deaths was considerably smaller than the present study.

As observed in the present study as well as previously, the strongest associations for cigarette smoking among both nondaily and daily smokers were observed for deaths from lung cancer and respiratory disease, with weaker but still important associations observed for deaths from cardiovascular and other diseases.^1,8,13,14^

### Strengths and Limitations

The most important strength of the present study is the detailed data on cigarette smoking patterns, which enabled the identification of lifelong nondaily smokers and the assessment of the mortality risks of reducing the level of smoking from daily to nondaily. We were also able to perform a sensitivity analysis excluding ever users of other tobacco products. Large sample size and nationally representative sample are also key strengths. With data collected from more than 500 000 adults, we were able to evaluate a dose-response association across detailed categories of number of cigarettes per month among lifelong nondaily smokers. Appropriate survey weights were applied so that findings from this study are representative of the US civilian, noninstitutionalized adult population.

The present study also has limitations. Cigarette smoking was assessed at 1 time point; therefore, smoking status may have changed during the follow-up. We relied on participants recalling their smoking, and approximately 20% of the questionnaires were completed by proxies. Thus, there is potentially recall bias.^15^ However, previous studies have shown that self-reported smoking, both daily and nondaily, is reliable and valid with good correlation with biomarkers such as nicotine and its metabolites.^16,17,18^ The surveys included in our study did not assess e-cigarette use; therefore, we were unable to examine the mortality risks of nondaily cigarette smokers who additionally use e-cigarettes and compare them with those who did not use e-cigarettes. Regarding other tobacco products, we restricted our sensitivity analysis to exclusive nondaily smokers who did not use pipe, cigar, or smokeless tobacco and found little change. Our study also lacked information on certain potential confounders, such as diet, physical activity, and medical history. However, the adjustment for physical activity or diet did not affect the association between smoking and mortality considerably in previous studies.^8,19^ Nevertheless, uncontrolled and residual confounding are potential limitations, as in any observational studies.

Nondaily smoking is more common among non-Hispanic black and Hispanic individuals than non-Hispanic white individuals. We found generally similar associations for daily and nondaily smoking across examined racial/ethnic groups; although our study is large, our sample size was relatively low for certain comparisons, such as mortality risks of lifelong nondaily smoking among Hispanic individuals. Future studies will inform us with the long-term patterns of nondaily smokers in the US population and possible differences by race/ethnicity, sex, and birth cohort.

Reflecting the US population, daily and nondaily smokers started smoking as teenagers in our study. Thus, observed mortality risks are for long-term daily and nondaily smokers. Because smoking duration, independent of intensity, is a particularly important determinant of disease risk,^20,21,22^ studies of nondaily smoking in other populations where people initiate smoking at older ages will also be informative.

## Conclusions

In this nationally representative study, both daily and nondaily smokers had substantially higher mortality risks than never smoking. Associations were observed among daily smokers who smoked 1 cigarette per day and among nondaily smokers who smoked 6 to 10 cigarettes per month, with risks among both groups increasing with greater use. Furthermore, the mortality risks of daily smokers who substantially reduced their cigarette consumption to become nondaily smokers decreased but remained elevated. Our findings indicate that even very low levels of cigarette smoking are hazardous, supporting public health recommendations that there is no safe level of smoking and that all smokers, including nondaily and very low-intensity smokers, should quit.

## References

[1] National Center for Chronic Disease Prevention and Health Promotion (US) Office on Smoking and Health The Health Consequences of Smoking—50 Years of Progress: A Report of the Surgeon General. Centers for Disease Control and Prevention; 2014. 24455788

[2] Eriksen M, Mackay J, Ross H The Tobacco Atlas. 6th ed American Cancer Society/World Lung Foundation; 2012.

[3] Jamal A, King BA, Neff LJ, Whitmill J, Babb SD, Graffunder CM Current cigarette smoking among adults—United States, 2005-2015. MMWR Morb Mortal Wkly Rep. 2016;65(44):1205-1211. doi:10.15585/mmwr.mm6544a2 27832052

[4] Creamer MR, Wang TW, Babb S, Tobacco product use and cessation indicators among adults—United States, 2018. MMWR Morb Mortal Wkly Rep. 2019;68(45):1013-1019. doi:10.15585/mmwr.mm6845a2 31725711 PMC6855510

[5] Shiffman S Light and intermittent smokers: background and perspective. Nicotine Tob Res. 2009;11(2):122-125. doi:10.1093/ntr/ntn020 19246630 PMC2658906

[6] Shiffman S, Paty JA, Gnys M, Kassel JD, Elash C Nicotine withdrawal in chippers and regular smokers: subjective and cognitive effects. Health Psychol. 1995;14(4):301-309. doi:10.1037/0278-6133.14.4.301 7556033

[7] Amrock SM, Weitzman M Adolescents’ perceptions of light and intermittent smoking in the United States. Pediatrics. 2015;135(2):246-254. doi:10.1542/peds.2014-2502 25583910 PMC4306801

[8] Inoue-Choi M, McNeel TS, Hartge P, Caporaso NE, Graubard BI, Freedman ND Non-daily cigarette smokers: mortality risks in the U.S. Am J Prev Med. 2019;56(1):27-37. doi:10.1016/j.amepre.2018.06.025 30454906 PMC7477821

[9] The Tobacco Use Supplement to the Current Population Survey. National Cancer Institute, Division of Cancer Control & Population Sciences. Accessed May 2, 2020. https://cancercontrol.cancer.gov/brp/tcrb/tus-cps/

[10] Christensen CH, Rostron B, Cosgrove C, Association of cigarette, cigar, and pipe use with mortality risk in the US population. JAMA Intern Med. 2018;178(4):469-476. doi:10.1001/jamainternmed.2017.8625 29459935 PMC5876825

[11] Luoto R, Uutela A, Puska P Occasional smoking increases total and cardiovascular mortality among men. Nicotine Tob Res. 2000;2(2):133-139. doi:10.1080/713688127 11072451

[12] Løchen ML, Gram IT, Mannsverk J, Association of occasional smoking with total mortality in the population-based Tromsø study, 2001-2015. BMJ Open. 2017;7(12):e019107. doi:10.1136/bmjopen-2017-019107 29288187 PMC5770901

[13] Carter BD, Abnet CC, Feskanich D, Smoking and mortality—beyond established causes. N Engl J Med. 2015;372(7):631-640. doi:10.1056/NEJMsa1407211 25671255

[14] Inoue-Choi M, Liao LM, Reyes-Guzman C, Hartge P, Caporaso N, Freedman ND Association of long-term, low-intensity smoking with all-cause and cause-specific mortality in the National Institutes of Health–AARP Diet and Health Study. JAMA Intern Med. 2017;177(1):87-95. doi:10.1001/jamainternmed.2016.7511 27918784 PMC5555224

[15] Soulakova JN, Bright BC, Crockett LJ On consistency of self- and proxy-reported regular smoking initiation age. J Subst Abus Alcohol. 2013;1(1):1001. 25408943 PMC4233135

[16] Shiffman S, Dunbar MS, Benowitz NL A comparison of nicotine biomarkers and smoking patterns in daily and nondaily smokers. Cancer Epidemiol Biomarkers Prev. 2014;23(7):1264-1272. doi:10.1158/1055-9965.EPI-13-1014 24740202 PMC4621008

[17] Brigham J, Lessov-Schlaggar CN, Javitz HS, McElroy M, Krasnow R, Swan GE Reliability of adult retrospective recall of lifetime tobacco use. Nicotine Tob Res. 2008;10(2):287-299. doi:10.1080/14622200701825718 18236293

[18] Caraballo RS, Giovino GA, Pechacek TF, Mowery PD Factors associated with discrepancies between self-reports on cigarette smoking and measured serum cotinine levels among persons aged 17 years or older: Third National Health and Nutrition Examination Survey, 1988-1994. Am J Epidemiol. 2001;153(8):807-814. doi:10.1093/aje/153.8.807 11296155

[19] Thun MJ, Apicella LF, Henley SJ Smoking vs other risk factors as the cause of smoking-attributable deaths: confounding in the courtroom. JAMA. 2000;284(6):706-712. doi:10.1001/jama.284.6.706 10927778

[20] Doll R, Peto R Cigarette smoking and bronchial carcinoma: dose and time relationships among regular smokers and lifelong non-smokers. J Epidemiol Community Health (1978). 1978;32(4):303-313. doi:10.1136/jech.32.4.303 744822 PMC1060963

[21] Flanders WD, Lally CA, Zhu BP, Henley SJ, Thun MJ Lung cancer mortality in relation to age, duration of smoking, and daily cigarette consumption: results from Cancer Prevention Study II. Cancer Res. 2003;63(19):6556-6562. 14559851

[22] Lubin JH, Caporaso NE Cigarette smoking and lung cancer: modeling total exposure and intensity. Cancer Epidemiol Biomarkers Prev. 2006;15(3):517-523. doi:10.1158/1055-9965.EPI-05-0863 16537710

